# American Association for Emergency Psychiatry Task Force on Medical Clearance of Adults Part I: Introduction, Review and Evidence-Based Guidelines

**DOI:** 10.5811/westjem.2016.10.32258

**Published:** 2017-01-19

**Authors:** Eric L. Anderson, Kimberly Nordstrom, Michael P. Wilson, Jennifer M. Peltzer-Jones, Leslie Zun, Anthony Ng, Michael H. Allen

**Affiliations:** * University of Maryland, Department of Psychiatry, College Park, Maryland; † University of Colorado School of Medicine, Department of Psychiatry, Aurora, Colorado; § Denver Health Medical Center, Emergency Psychiatry, Denver, Colorado; ¶ Department of Emergency Medicine Behavioral Emergencies Research lab, University of Arkansas for Medical Sciences, Little Rock, Arkansas; || Henry Ford Hospital, Senior Staff Psychologist, Department of Emergency Medicine, Detroit, Michigan; # Chicago Medical School, Department of Emergency Medicine, North Chicago, Illinois; ** Uniformed Services School of Medicine, Department of Psychiatry, Bethesda, Maryland

## Abstract

**Introduction:**

In the United States, the number of patients presenting to the emergency department (ED) for a mental health concern is significant and expected to grow. The breadth of the medical evaluation of these patients is controversial. Attempts have been made to establish a standard evaluation for these patients, but to date no nationally accepted standards exist. A task force of the American Association of Emergency Psychiatry, consisting of physicians from emergency medicine and psychiatry, and a psychologist was convened to form consensus recommendations on the medical evaluation of psychiatric patients presenting to EDs.

**Methods:**

The task force reviewed existing literature on the topic of medical evaluation of psychiatric patients in the ED (Part I) and then combined this with expert consensus (Part II).

**Results:**

In Part I, we discuss terminological issues and existing evidence on medical exams and laboratory studies of psychiatric patients in the ED.

**Conclusion:**

Emergency physicians should work cooperatively with psychiatric receiving facilities to decrease unnecessary testing while increasing the quality of medical screening exams for psychiatric patients who present to EDs.

## INTRODUCTION

Psychiatric disorders are second only to cardiovascular disease as the leading cause of lost productivity in the United States.^1^ From 1992 to 2001, 53 million visits to the emergency department (ED) were for psychiatric complaints, a rate of 4.9%–6.3% of all ED visits,^2^ with 3.6% receiving a mental disorder diagnosis at discharge.^3^ When substance abuse is added to mental health disorders, one survey found the combined rate to be 12.5% over a year.^4^

EDs have become the primary and acute healthcare providers for many with mental health problems. Given recent legislation, the closure of state institutions, the national shortage of psychiatrists, reductions in funding for community mental healthcare including community-based crisis services, and dwindling numbers of inpatient psychiatric beds, the number of psychiatric patients presenting to EDs is increasing and likely to continue.^1^,^5^–^6^ As a result of these and other factors, psychiatric emergency visits are resource-intensive, longer and may contribute to crowding as well.^7^–^9^

There are four common reasons for patients who present with psychiatric concerns to receive a medical assessment. First, patients may have medical problems that are the primary cause of the presentation and require care exclusively in a medical setting. Second, medical problems may complicate or contribute significantly to a psychiatric problem such that medical care takes precedence and may obviate the need for psychiatric care. Third, medical problems may be completely coincidental but require attention during confinement for psychiatric care. Fourth, there may be medical problems that, under other circumstances, might be deferred indefinitely but cannot be neglected by the mental health facility to which the patient is transferred. The rationale for and execution of medical screening for each of these situations varies by location, yet they are all subsumed under the rubric of “medical clearance.”

Few would argue about the necessity of careful screening in the first two situations above. However, the complexity of the screening is further modified by the capabilities of psychiatric receiving facilities, as they vary in their ability to assess and treat medical problems.^6^ This often shifts the burden for the seemingly routine medical assessment and treatment planning in the last two situations above to emergency services. While the problems associated with the first two are more susceptible to scientific debate, the problems of the second two often have more to do with payment mechanisms and health policy.

In Part I of this series, an American Association for Emergency Psychiatry (AAEP) Task Force provides an overview of medical assessment of psychiatric patients in the ED, including review of the literature and evidence-based guidelines. In Part II of the series, the task force discusses controversies in medical clearance and presents an AAEP consensus statement on medical assessment. Selected articles were chosen individually by committee members on the basis of their relevance to the medical screening process. Existing medical screening policies, such as the one by the American College of Emergency Physicians (ACEP), were also carefully reviewed. Task force members consisted of Eric L. Anderson, Kimberly Nordstrom, Michael P. Wilson, Jennifer M. Peltzer-Jones, Leslie Zun, Anthony Ng, and Michael H. Allen chosen by the AAEP for their expertise on the topic, all with an extensive background in behavioral emergencies.

## TERMINOLOGY PROBLEMS

Perhaps the first major hurdle in creating a consensus is agreement upon terminology. Depending upon how the term is used, “medical clearance” may imply patient readiness for psychiatric evaluation, stability for transfer to inpatient psychiatry, or stability for discharge to outpatient care. Additionally, depending upon the level of care to which the patient is referred, more or less stringent evaluation may be required. Some facilities have ready access to medical and surgical services and thus are better prepared to accept medically complex patients. Other facilities, especially freestanding psychiatric hospitals, often do not have easy access to medical and surgical services. Psychiatric patients with complex medical problems may not be within their capabilities, despite having originally presented with a psychiatric complaint.^10^

According to Weissberg (1979), “medical clearance” itself is an imprecise term that implies “everything has been done and no problems have been found.”^11^ There are at least three situations where the term is often used: 1) No medical condition is thought to be present; 2) a medical condition, e.g., hypertension, is known but is not thought to be the primary cause of psychiatric symptoms; and 3) a medical condition, e.g., intoxication, was present but no longer needs treatment. The term is often used to imply that causative medical problems have been excluded. Confusion may thus arise with the term “medical clearance,” and some authors have suggested that the term be replaced with a more precise description, such as the narrative of the patient’s clinical condition.^12^

Traditional or historic terms, as well as current and preferred terms, are presented in Table 1. In the following reviews of the literature, the original language was maintained for fidelity’s sake.

## MEDICAL ILLNESS IN PSYCHIATRIC PATIENTS

Medical problems are common in patients with psychiatric diagnoses.^13^ Psychiatric presentations usually require some form of medical as well as psychiatric assessment.^14^–^17^ Although the extent to which medical illness contributes to psychiatric symptoms has been the subject of much debate and research, medical illness is prevalent in mentally disordered patients.^18^–^26^ In studies of psychiatric patients, Hall et al found that as many as 46% of patients had a medical etiology for their symptoms.^20^–^21^ Similar results were found by Koranyi (1979), who found 43% of patients with at least one physical illness;^22^ Summers et al (1981) who reported a medical illness frequency of 33.5%–63%;^23^ Carlson et al (1981) who reported a frequency of 75%;^24^ Olshaker et al (1997) who reported incidence of 24%–50%;^25^ and Zun et al (1996) who reported an incidence of 19%–75%.^26^ In 1994, Tintinalli et al reported that, in 80% of patients for whom a medical diagnosis should have been made, a “medically clear” label was given.^27^ Taken together, the rate of comorbid medical illness that may contribute to, exacerbate, or cause any given patient’s psychiatric symptoms ranges from 19 to 80%, but the true incidence is difficult to ascertain given the limitations of many of these studies, such as a lack of follow up, potential selection bias, and convenience sampling.

While the precise extent to which medical mimics of psychiatric disease are misdiagnosed as mental illness is unknown, it may be fairly common. For example, a study by Han et al (2009) found that delirium was common in the ED and that emergency physicians (EP) missed the diagnosis in 76% of cases.^18^ Hustey et al (2003) found that impairment in mental status was 27% in their sample of ED patients, but that EPs altered their management in zero cases when informed.^28^ The consequences of misdiagnosis may be grave. Hoffman for instance reported that 63% of patients originally admitted for dementia were found to have a treatable condition, and Reeves et al (2010) found that elderly patients with delirium who were admitted to psychiatric units were less likely to undergo complete diagnostic assessments than delirious elderly patients admitted to medical units.^29^–^30^

## REVIEW OF THE LITERATURE

Hall et al (1981) examined 100 consecutive admissions to a research inpatient psychiatric unit and found that, with extensive testing, medical problems could be identified in 46% of patients.^21^ They recommended, as routine screening, a complete psychiatric history, detailed neurological examination, 34-panel chemistry, electrocardiogram (EKG), complete blood count (CBC), urinalysis, and a sleep-deprived electroencephalogram (EEG) for new onset psychiatric symptoms. Dolan et al (1985), on the other hand, examined the clinical utility of routine laboratory testing in 785 patients in a psychiatric hospital. They found that clinically important abnormal findings were uncommon (4% of their study population),^31^ consistent with Korvin et al (1975), who found only 223 clinically significant laboratory findings in a sample of 19,980 test results (a rate of 1.1%).^32^ As with prior studies, these were limited by convenience sampling rather than random assignment of subjects. Detailed screening is associated with more consultations, more diagnostic investigations, and higher costs.^33^

Henneman et al (1994) evaluated a standardized ED medical evaluation conducted in 100 patients with new-onset psychiatric symptoms;^34^ 63% had an “organic” etiology. They recommended routine, comprehensive laboratory screening as an integral part of the medical evaluation of alert patients with new psychiatric symptoms. In contrast, Olshaker et al (1997) evaluated the frequency of medical conditions in 345 patients in a retrospective study over a two-month period.^25^ They found that 19% had medical conditions, most of which were identified via the history, physical exam (PE), and vital signs. They concluded that routine laboratory tests, including CBC, chemistry panels, and toxicology screening had a low yield.

Korn et al (2000) reported that comprehensive screening of all patients is prohibitive and an unnecessary use of resources.^35^ In a retrospective chart review, they found that 38% of all patients had isolated psychiatric complaints and 62% had both medical and psychiatric complaints. They recommended routine laboratory examination for patients with substance abuse, the elderly, homeless, and patients with new symptoms. They recommended against laboratory studies in patients with an established psychiatric history who had no medical complaints, no PE findings, and stable vital signs. This study was limited in that it was retrospective and only reviewed data over a five-month period.

A retrospective review of charts of those who were admitted to a psychiatric ED who had been expected to undergo a medical clearance process, found a wide variation in the PEs done in the ED by EPs, psychiatric residents/students and family practice (FP) physicians or FP nurse practitioners. In this study, FP physicians and nurses had the most complete exams, while EPs had the least complete exam.^36^

Although mental health patients in the emergency setting are sometimes assumed to have difficulty reporting medical symptoms or history accurately, Amin and Wang (2009) argued that no literature supports this view,^37^ and at least some researchers have argued the complementary point that patients have a desire to be treated as credible reporters.^38^ In the Amin and Wang study of 375 patients, only four had significant lab abnormalities that did not lead to any change in their disposition.^37^ The authors concluded that the history and PE is sufficient in patients with psychiatric complaints for whom there is documentation of previous psychiatric history and a normal history and PE.

To clarify the importance of a history and physical, Reeves et al (2000) correlated physical findings with medical diagnoses in a group of psychiatric patients and found failure to obtain available history in 34.4%, an inadequate PE in 43.8%, and an inadequate mental status examination in 100% of those with missed medical diagnoses.^39^ However, the population in their study was small (n=64). Further, in a sample of 1,340 patients admitted to a psychiatric unit between 2001 and 2007, Reeves et al (2010) found that a medical disorder had caused the symptoms of 55 patients (2.8%). Compared to patients admitted to medical units, patients admitted to psychiatric units had lower rates of completion of medical histories, PEs, cognitive assessments, indicated laboratory and/or radiologic studies, and treatment of abnormal vital signs. The authors concluded that assessment procedures are less likely to be performed in patients admitted to psychiatric units with mental status changes because the symptoms are more likely to be attributed to psychiatric illness than are those of patients without such a history.^40^

Given the conflicting literature on the utility of universal screening, it is perhaps not surprising that this is often an area of disagreement between EPs and psychiatrists. Broderick et al (2002) for instance reported that universal, as opposed to indicated, laboratory screening was one of the greatest barriers to consensus between the ED and psychiatry with respect to the medical examination.^41^

Substance abuse may be an indication for more extensive medical assessment but the screening method required is also controversial. In their 2000 study of 392 patients who presented to a psychiatric emergency service, Schiller et al found routine urine drug screening did not have an appreciable impact on either patient disposition or length of inpatient stay. The authors concluded that routine use of drug screening in such settings was not supported by their results.^42^

Agitation may also be an indication for further testing. Schillerstrom et al (2004) found several laboratory differences between agitated patients who required emergent medication and non-agitated patients. The authors concluded agitated psychiatric patients may be medically different from non-agitated patients and argued for testing. Limitations of their study included a short data collection period, retrospective design, and inconsistent measurements between subjects.^43^

In a review paper, Gregory et al noted that psychiatric patients in the ED should undergo screening if they are considered for a psychiatric admission.^10^ The screening is intended to identify patients who cannot be safely or effectively treated on a psychiatric unit. Accordingly, medical clearance does not mean the patient is free of illness, but that there is no acute need to transfer the patient to a medicine service. The authors highlighted the need for greater standardization and provided a sample protocol for medical screening examinations.

Based upon a thorough review of the medical literature regarding medical assessment of psychiatric patients in 2005, Zun et al concluded that new-onset psychiatric symptoms require extensive ED evaluation but patients with chronic psychiatric illnesses do not need routine testing if the presentation was similar to past presentations. They also suggested that documentation of the medical assessment has more value than use of the ambiguous term “medically clear.”^12^

Janiak et al (2010) noted that psychiatric treatment facilities have varying requirements for baseline testing and interventions before accepting patients. They argued that the history and PE performed by the ED is sufficient to identify medically compromised patients, and that tests done per psychiatric protocol are not cost-effective. However, the psychiatric service in their study had ready access to medical consultation and treatment services if needed, which is not the case in many free-standing psychiatric hospitals.^44^

Of note, requirements of the Emergency Medical Treatment and Labor Act (EMTALA) have at times been confused with what psychiatric facilities consider “medical clearance.” There is a commonly held belief that if the ED does not complete a full medical clearance, there is risk of an EMTALA violation. However, under EMTALA 1) any individual who comes to an ED and requests care must receive a medical screening examination to determine whether an emergency medical condition exists; and 2) if an emergency medical condition exists, treatment must be provided until the emergency medical condition is resolved or stabilized. This is not the same thing as “medical clearance” but rather stabilization of emergency conditions. There is no difference when it comes to a psychiatric condition; stabilization or transfer to a higher level of care must occur. There is no requirement for “universal” laboratory tests to be completed. This has led to disagreements between hospitals and disciplines. A free-standing psychiatric facility may feel compelled to reject the patient on grounds of medical stability, when in fact, the issue may be very different.

In summary, the best available evidence indicates that a thorough history and PE, including vital signs, are the minimum necessary elements in the evaluation of psychiatric patients. However, this has never been specifically studied in a randomized clinical trial.

## AVAILABLE PROTOCOLS AND GUIDELINES

Several efforts have been made to standardize the evaluation of psychiatric patients. It is worth noting that efforts to create guidelines are often met with resistance from both mental health professionals and EPs.^45^–^46^

In 1996, Zun et al developed a tool to evaluate the appropriateness of patient transfer to state psychiatric beds.^47^–^48^ This protocol reduced costs, but did not reduce throughput or ED rates of recidivism. In a 2010 report, Pinto et al noted that the “goal of medical clearance” is to determine if medical illnesses make admissions to psychiatry inappropriate or unsafe. They provided a template for the PE of psychiatric patients,^36^ but clinical trials of the template are lacking.

Shah et al (2010) developed a two-part screening tool and retrospectively examined 500 charts of patients for whom psychiatric symptoms were the chief complaint. They concluded that their screening tool could be used to identify patients who can be referred for psychiatric evaluation without laboratory tests.^49^

Multiple states and hospitals have also developed tools and protocols for the evaluation of psychiatric patients in an effort to cut costs, enhance throughput, standardize evaluation, and improve patient care. Examples of these include the Maine Health Medical Clearance Protocol,^50^ Massachusetts College of Emergency Physicians: Joint Task Force Consensus Guidelines,^51^ North Carolina Department of Mental Health guidelines (revised),^52^ and University of Connecticut Health Center Medical Clearance protocol.^53^ (See Table 2.) Unfortunately, few data are available concerning validation of these protocols.

In 2006, the Clinical Policy Committee of the American College of Emergency Physicians introduced a policy for evaluation of psychiatric patients presenting in the ED based on an extensive review of the literature.^54^ Patients with abnormal vital signs, delirium, altered cognition, or abnormal physical examinations were excluded “because they often have medical illness that mandates a symptom-based evaluation.” Several conclusions were offered by the task force with respect to the medical assessment process: 1) In alert, cooperative patients with normal vital signs, a noncontributory history and PE, and psychiatric symptoms, routine laboratory testing was felt to be of low yield and not necessary; 2) In alert, cooperative patients with normal vital signs, a noncontributory history and PE, and psychiatric symptoms, routine urine toxicology need not be performed, and screens obtained for the use of receiving psychiatric facilities should not delay the patient’s evaluation or transfer; and 3) In alert, cooperative patients with normal vital signs, a noncontributory history and PE, and an elevated blood alcohol level, the patient’s cognitive abilities rather than a specific blood alcohol level should be the basis upon which to begin a psychiatric assessment.

## CONCLUSION

The review of the medical screening literature is varied, with multiple studies, multiple authors, and multiple methodologies used to investigate this question. Perhaps given the variability in study designs and populations, the literature is rife with controversy. The next article will present consensus recommendations in an effort to establish nationally accepted guidelines.

## Figures and Tables

**Table 1:** Terminology of historic/literature terms.

Term	Definition
Medical clearance	A general name for the process of ensuring the patient does not have a medical condition that requires further attention. It does not provide any guidance as to the purpose or depth of the evaluation, nor does it define the role of any medical conditions, if present. It implies a follow-on action, i.e., clearance to do something else, such as transfer or discharge the patient.
Medically clear	A term meaning that, in the opinion of the examining provider, the patient does not have any medical condition which merits further treatment or concern.
Medical assessment	A general name for the process of examining a patient for active or pertinent medical conditions. Unlike medical clearance, it does not imply any particular downstream goal.
Medical evaluation	A term that generally means the same thing as medical assessment.
Medical screening	Closely related to medical assessment and medical evaluation, screening usually implies that specific issues are being sought for presence or absence.
Organic clearance	A term that describes the process of eliminating somatic, non-psychological reasons for the patient’s symptoms (although arguably most Axis I diagnoses have an organic etiology and/or pathogenesis, but these mechanisms have not been fully elucidated).
Focused evaluation/examination	A term that implies an evaluation of smaller scope than assessments, evaluation, or clearance.
Preferred/current terms causal, contributory, and/or incidental	These define the presence of medical condition(s), and whether those conditions have led to the current presentation, contributed to it, or were just found in the process of evaluating the patient
Stable vs. unstable	This more succinctly defines the status of the patient, regardless of the contribution of any medical conditions, and their appropriateness for discharge or transfer to another level of care

**Table 2:** Medical clearance as currently practiced in select states.

	Clearance?	Labs	Should not admit	BAL/UDS	Special notes
University of Connecticut	Performed by ED	Per HPI/physical exam; some labs required for patients presenting for detox, overdose, or eating disorders	Patients on O2 therapy; who require IVs; who have high acuity; who require telemetry	BAL on all patients for detox; UDS on patients with overdose	Patients with BAL > 100 should stay in the ED
Massachusetts College of Emergency Physicians	Reflects short-term but not long-term medical stability. Does not indicate the absence of ongoing medical issues	Not required for low-risk patients (age 15–55, no acute complaints, no new psychiatric or physical symptoms, no substance use, normal physical exam, normal vitals)	Not specified	Neither the determination that the patient can be psychiatrically evaluated nor the determination that a patient can be transferred should be based on a specific level of alcohol	ED exam is focal and not a replacement for a general multisystem physical exam after transfer. Additional testing may be performed if receiving facility asks for it, but should not delay transfer.
Best practices report/Illinois Hospital Association	Focused medical assessment by ED preferred over term “medical clearance”	Not required if patient has no new psychiatric condition, no hx of active medical illness, normal vitals, normal physical exam, normal mental status	Not specified	Patient cannot be assessed psychiatrically if intoxicated, but cognitive abilities rather than absolute level should guide assessment.	If intoxicated, patient should remain in the ED. This is not a function of a specific alcohol level.
North Carolina	Performed by ED	Not required for low-risk patients	NC psych facilities cannot safely manage serious medical conditions, such as (see report for full list): transfusions; recent head injury without workup; CVA; recent MI requiring telemetry; hypertensive crisis; acute drug intoxication; acute fracture; unexplained fever; DKA	BAL should be <300	Pay special attention to elderly patients, as medications may be causing their symptoms

*ED,* emergency department; *HPI,* history of present illness; *BAL,* blood alcohol level; *UDS,* urine drug screen; *NC,* North Carolina; *CVA,* cerebrovascular accident; *MI,* myocardial infarction; *DKA,* diabetic ketoacidosis
